# Our successful surgical coronary revascularization in a case with familial Mediterranean fever

**DOI:** 10.1186/1749-8090-8-S1-P91

**Published:** 2013-09-11

**Authors:** U Yetkin, S Yazman, H Iner, I Yurekli, O Gokalp, A Gurbuz

**Affiliations:** 1 Department of Cardiovascular Surgery, Izmir Katip Celebi University Ataturk Training and Research Hospital, Izmir, Turkey

## Background

Familial Mediterranean fever (FMF) is an autosomal recessive genetic disorder. The diagnosis of this disorder is based on clinical symptoms. This disorder is important epidemiologically for our country. Therefore, many difficulties are experienced in terms of making definitive diagnosis and therapeutic approaches in additional pathologies.

## Methods

Our case was a 56-year-old male. His past medical history was significant for coronary artery disease with stenting of left anterior descending artery 5 months ago and FMF diagnosed 2 years ago in remission state with colchicine therapy. He was suffering from chest pain for 4 months and coronary angiogram showed an in-stent stenosis of 90%. He was hospitalized for surgical revascularization. Transthoracic echocardiography showed a left ventricular ejection fraction of 50% and a mean pulmonary arterial pressure of 35 mmHg.

## Results

Preoperative consultation with Rheumatology Department stated that it was not an active episode of the disorder and no drawback was identified for operation. He was then taken into the operating room. Periaortic adhesions were dissected carefully. LIMA-LAD and Ao-SVG-Diagonal bypasses were carried out. Postoperative period was event-free. He was still under close follow-up of both departments.

## Conclusions

We did not find the erysipeloid erythematous lesions typical for FMF in the lower extremities; thus, we harvested saphenous vein for autogenous conduit. Exacerbations of fever – which is also characteristic but not specific for FMF - was not detected during postoperative period. Cardiac surgeons should be aware of the complications of such chronic disorders with exacerbations that may affect the perioperative prognosis.

